# Ring Oscillators with Additional Phase Detectors as a Random Source in a Random Number Generator

**DOI:** 10.3390/e27010015

**Published:** 2024-12-28

**Authors:** Łukasz Matuszewski, Mieczysław Jessa, Jakub Nikonowicz

**Affiliations:** Faculty of Computing and Telecommunications, Poznań University of Technology, 60-965 Poznań, Poland; mieczyslaw.jessa@put.poznan.pl (M.J.); jakub.nikonowicz@put.poznan.pl (J.N.)

**Keywords:** random number generator, randomness, ring oscillators, entropy, restarts, statistical tests, FPGAs

## Abstract

In this paper, we propose a method to enhance the performance of a random number generator (RNG) that exploits ring oscillators (ROs). Our approach employs additional phase detectors to extract more entropy; thus, RNG uses fewer resources to produce bit sequences that pass all statistical tests proposed by National Institute of Standards and Technology (NIST). Generating a specified number of bits is on-demand, eliminating the need for continuous RNG operation. This feature enhances the security of the produced sequences, as eavesdroppers are unable to observe the continuous random bit generation process, such as through monitoring power lines. Furthermore, our research demonstrates that the proposed RNG’s perfect properties remain unaffected by the manufacturer of the field-programmable gate arrays (FPGAs) used for implementation. This independence ensures the RNG’s reliability and consistency across various FPGA manufacturers. Additionally, we highlight that the tests recommended by the NIST may prove insufficient in assessing the randomness of the output bit streams produced by RO-based RNGs.

## 1. Introduction

Random number generators (RNGs) are crucial in numerous applications [1]. These generators fall into two main categories: pseudo-random number generators (PRNGs), characterized by a deterministic algorithm, and true random number generators (TRNGs), capable of generating non-deterministic sequences even when the generator’s structure is known. In the current digital-centric environment, there is a growing expectation for true random number generators to fully integrate into a single chip within systems that rely on random numbers. This integration is particularly pertinent in cryptographic systems where the complexity of retrieving and manipulating bits or sequences produced by TRNGs adds an extra layer of security. To address security concerns, we propose introducing a start–stop mechanism to enable the on-demand generation of a specified number of random bits. This approach disrupts real-time observation of the random bit production process, presenting a formidable challenge to potential attackers attempting to exploit vulnerabilities, such as monitoring the supply system.

Among the most widely adopted solutions for entirely digital RNGs, easily implementable in field-programmable gate arrays (FPGAs) or application-specific circuits (ASICs) and compatible with various cryptographic systems are those founded on the principles of jitter in ring oscillators or on metastable states [2,3,4,5,6,7,8,9,10,11,12,13,14,15,16,17,18,19,20,21,22,23,24,25,26].

The primary limitation of conventional RNGs lies in the necessity for the post-processing of output bits. This stems from the inherent bias and strong correlation among adjacent bits in the output sequences, causing them to be unable to pass numerous statistical tests. To address this, a transformation, termed post-processing, is applied to enhance statistical properties, albeit at the expense of reducing the bit rate. Since statistical tests lack the capability to differentiate between deterministic and non-deterministic generators—both PRNGs and TRNGs can satisfy these tests—an additional mechanism is required to ascertain the origin of the output numbers.

Distinguishing between deterministic and non-deterministic sources becomes challenging due to the presence of deterministic and non-deterministic components in RNG signals [14,15,16,17]. A notable instance is the RNG proposed by Wold and Tan [4], a modification of the Sunar et al. RNG, exploiting jitter in ring oscillators [3]. Wold and Tan introduced an additional flip-flop to the ring oscillator’s output preceding the XOR gate. This modification allowed the output bit streams to pass statistical tests without further transformations and with a considerably reduced number of ring oscillators compared to the original proposition. Subsequent optimization by Wold and Petrović led to reported speeds of producing random bits in an FPGA reaching 300 Mbit/s [5]. In [27], Bouchard et al. demonstrated through simulation experiments that Wold and Tan’s method can yield sequences passing all statistical tests even without jitter in ring oscillators. Consequently, the robust statistical properties of bit streams generated by Wold and Tan’s combined RNG can be achieved using a fully deterministic source. Therefore, evaluating the degree of true randomness is imperative before embracing a particular solution as a TRNG.

Such an evaluation can be conducted directly by measuring the jitter in the signals produced by RO, using tools such as oscilloscopes or dedicated devices [14,18]. Alternatively, a different measure can be considered, as exemplified by the concept of restarts [28,29]. With this method, randomness is indirectly assessed by observing the characteristics of bit sequences generated during successive restarts of an RNG from the same initial conditions at different time points. Although restarts seem more applicable in field-programmable gate arrays (FPGAs), they are effective only for RO-based RNGs. ROs can initiate their operation from identical initial states of zero or one.

This paper presents an innovative design for an RO-based RNG inspired by Wold’s and Tan’s configurations. Our approach incorporates additional phase detectors, enhancing the randomness extraction from the ROs. To strengthen the security of the proposed RNG, we support the on-demand generation of bits. This involves initiating and stopping the RNG’s operation as required by a digital system implemented within the same FPGA or ASIC.

Section 2 provides a comprehensive overview of the proposed solution, detailing its construction and functionality. Section 3 reports the outcomes of rigorous tests and measurements. Section 4 describes the optimization of FPGA resources for implementing the proposed RNG. The paper concludes with a summary of the findings in Section 5.

## 2. Combined RNG with Additional Phase Detectors

**Figure 1:**
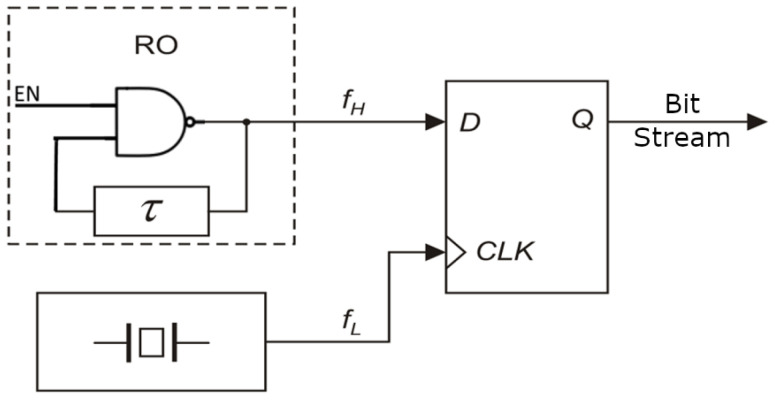
Jitter oscillator sampling method for producing random bits.

The operational principle of a single RO-based RNG with a start–stop function is depicted in Figure 1. The signal from the RO undergoes sampling in a flip-flop, utilizing another signal characterized by a lower frequency (Figure 1). Typically, the delay τ is implemented as a chain of even-number inverters, a series of latches, or alternatively, a delay line [30].

When EN= “1”, the RO generates rectangular waves sampled in the flip-flop, synchronized with a quartz oscillator signal. Conversely, the generator remains inactive when EN= “0”. This type of RNG can furnish a predetermined number of bits on demand, a parameter easily regulated by the number of impulses generated by the quartz oscillator.

**Figure 2:**
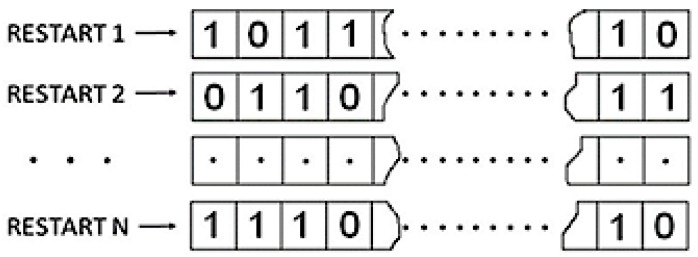
Successive restarts of RO-based RNG from Figure 1. During a single restart, an *M*-bit sequence is produced.

When the output signal of the RO consists solely of deterministic components, the binary sequences generated for each restart of the RNG remain identical. In cases where the level of randomness is substantial, the initial bits (m=1) of sequences produced during consecutive restarts exhibit an equal probability of 0.5, as illustrated by bits 10…1 in Figure 2.

If the output signal of RO contains only deterministic components, then binary sequences produced for all of the restarts of RNG are the same. If the amount of randomness is sufficient, then the first bits (m=1) of the sequences produced by successive restarts appear with the same probability equal to 0.5 (bits 10…1 in Figure 2).

The same concerns the second set of bits (m=2, represented by bits 01…1 in Figure 2), as well as subsequent sets, such as the third set (m=3, bits 11…1 in Figure 2), and so forth, where *m* is an arbitrary value less than *M*. In real circuit implementations of RO-based RNGs, an immediate pool of high-entropy random bits is not readily available. The non-deterministic jitter inherent in a single period of an RO signal is insufficient and needs to accumulate before being sampled. This accumulation often spans numerous cycles of the quartz oscillator. Consequently, zeros and ones in *N*-bit sequences exhibit equal probability after a relatively extended period, corresponding to relatively large values of *m* or, conversely, for very low sampling frequencies, on the order of several Hz [17].

For indices 1<j<m, *N*-bit sequences demonstrate bias, precluding the production of the required number of bits on demand using subsequent bits from the *M*-bit sequence. To ensure an equal probability of zeros and ones, one could select every *m*-th bit as the output bit, but this approach substantially diminishes the overall bit rate. For instance, an RNG based on a single RO yields only a few random bits per second [17], and theoretically, the 300 Mbit/s RNG proposed in [5] would generate less than 15 Mbit/s. This inherent limitation can be mitigated through the allocation of additional resources, such as post-processing techniques that leverage a block cipher operating in the Output Feedback Mode (OFB) or Counter Mode (CTR) or through the use of a hash function [1].

**Figure 3:**
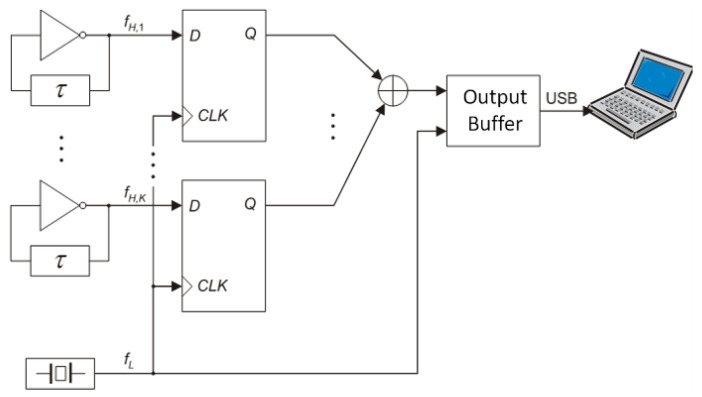
Successive restarts of RO-based RNG from Figure 1. During a single restart, an *M*-bit sequence is produced.

One established technique for reducing *m* and improving the statistical quality of bit sequences involves combining XOR bit streams generated by numerous independent RNGs, referred to here as elementary generators (see Figure 3) [4,5,28,29,30].

When the bias is zero, the combination of XOR always diminishes the covariance (correlation) between adjacent bits to zero [31]. This holds true when independent elementary generators generate the combined bit streams. Unfortunately, achieving complete independence is challenging when these elementary generators are implemented within the same digital circuit. Additionally, distinguishing between dependent and independent generators remains uncertain, and their interdependencies cannot be assessed during the bit stream generation process. By maliciously targeting a combined RO-based RNG, one can manipulate the number of dependent generators while reducing the count of independent ones, theoretically to zero, especially when the frequencies of the ROs closely match the frequency of the injected signal [13,32,33,34].

Two strategies can be applied to bolster resilience against such attacks. Firstly, ROs can be designed with significantly different nominal frequencies by altering the delay τ. This can be achieved, for example, by implementing a varying number of inverters, latches, or taps of delay lines for different ROs [30]. Secondly, increasing the number of elementary RO-based RNGs can be instrumental. Due to the distinct path lengths connecting components of the combined RNG in real circuits, a broad spectrum of RO frequencies can be obtained, posing a formidable challenge to potential attackers. Introducing a start–stop operation in the RNG can serve as an additional obstacle, but ensuring the independence of all bits in an *M*-element sequence is imperative. The evaluation of bit independence and the statistical quality of output sequences is conducted using dedicated test suites. Equally crucial is the generation of bits through a non-deterministic mechanism. Suppose a potent deterministic component exists in the signal. In that case, adversaries may exploit it to reduce the number of generated sequences or even coerce a random number generator into producing a sequence with easily predictable bits. Such manipulation may go unnoticed by the monitoring system since the sequences still pass statistical tests. Implementing restarts, and uniformity testing for subsequent *N*-bit sequences becomes essential to detect a deterministic component, preventing the production of sequences with easily predictable bits or groups of bits.

**Figure 4:**
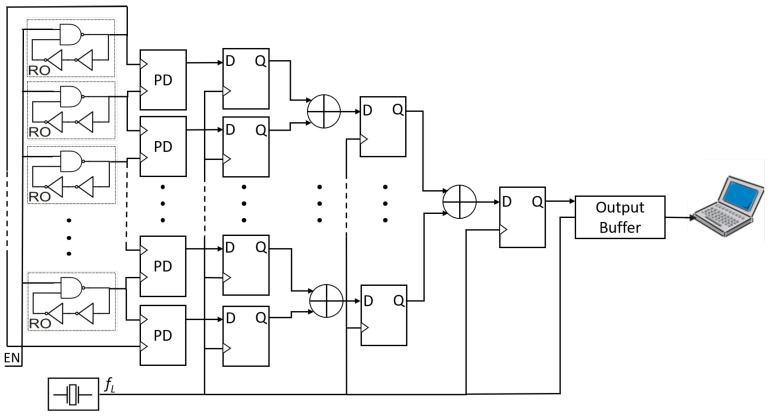
A start–stop combined RNG with phase detectors.

**Figure 5:**
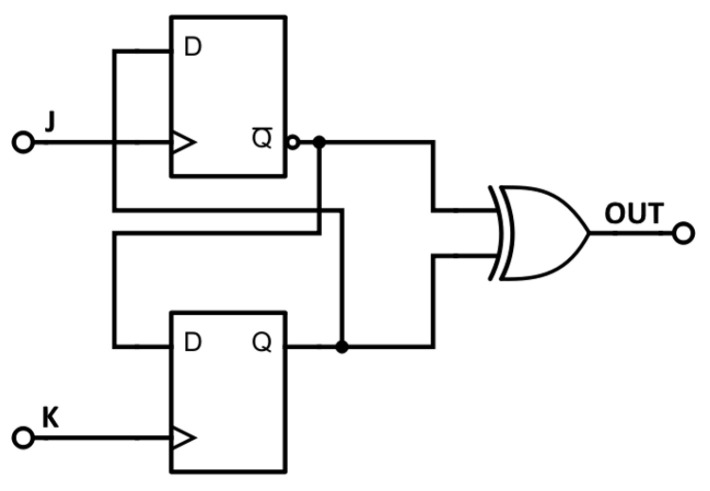
A scheme of the phase detector PD used in the start–stop combined RNG from Figure 4.

The most straightforward approach is to employ more ROs as entropy sources to enhance the harvested randomness. This method aligns with the proposal made by Sunar et al. Alternatively, we can increase entropy by augmenting the number, *K*, of elementary generators. In this paper, we opt for the latter solution, albeit modified with additional phase detectors. By harvesting more randomness, we effectively reduce *K*, which in turn lowers the resources needed for on-demand bit production, compared to existing approaches in the literature that rely on ROs. The block scheme of the proposed generator is illustrated in Figure 4, utilizing *K* ROs and *K* phase detectors (PDs). Each phase detector comprises two *D* flip-flops and one XOR gate, as depicted in Figure 5. This edge-type detector is not sensitive to the input duty cycle, as indicated in Table 1. The XOR gate combines bits produced by *K* elementary RNGs in a cascade, simultaneously sampling outputs with flip-flops. Given that the RNG is designed to operate on any FPGA or ASIC, no manual placement or routing adjustments were made during the implementation process. The RNG is engineered to function with a clock frequency exceeding 1 MHz. However, a single XOR gate or a series of Look-Up Tables (LUTs) simulating an XOR gate cannot effectively process numerous signals at high frequencies. To address this limitation, signals are grouped, and additional flip-flops are introduced. In contemporary FPGAs, the minimum number of LUT inputs is four.

The signals sampled from phase detectors are consolidated in a single LUT. Subsequently, the output bits are sampled by the same clock using a D flip-flop to ensure synchronization and to create a new set of source streams. These new source streams are further categorized into groups. Within the same group, the streams are XOR-ed, and the resulting bits are sampled by the same clock, generating the subsequent source stream. This process continues iteratively.

Although the ROs in the design employ an identical number of inverters, the frequencies of theoretically identical ROs exhibit significant variations (see Table 2). This discrepancy arises from distinct path lengths between logic gates after implementing ROs in the digital circuit. As noted in [5], the ring oscillator with three inverters demonstrates the highest dispersion in frequency, a finding consistent with our presented results in Table 2. The phase detector establishes a phase difference between adjacent ring oscillators. The phase can be regarded as a random value due to substantial jitter and frequency drift. This phase difference is then sampled using a D flip-flop triggered by a quartz oscillator, setting the output bit rate. The sequences from the flip-flops are subsequently XOR-ed. Utilizing a phase detector to ascertain the phase difference between adjacent ROs results in a signal with more significant phase fluctuations.

The jittered signal from a single RO can be expressed as a phase-modulated signal [35]:

(1) s(t)=P[ωt+φ(t)],

where *P* represents the sequence of periodic square pulses, *t* denotes time, ω=2πf denotes the pulsation of the periodic signal with frequency *f*, and φ accounts for phase fluctuations (jitter). Through Fourier series analysis of the s(t) signal, it can be demonstrated that the rectangular signal shares similar phase properties with its first harmonic [35]. This characteristic significantly simplifies the analysis of a signal with phase jitter. Therefore, a sinusoidal signal with jitter can be expressed as follows:

(2) s(t)=sin(ωt+φ(t)),

or

(3) s(t)=sin(ωt)cos(φ(t))+sin(φ(t))cos(ωt).

Due to the minimal magnitude of phase fluctuations, the cosine of small angles approximates one, and the sine approaches the angle value. Therefore, Equation (3) can be approximated as follows:

(4) s(t)=sin(ωt)+φ(t)cos(ωt).

The signal from the *k*-th ring oscillator, where k=0,1,2,…,K−1, is as follows:

(5) $$s_{k}\left(t\right)=\sin\left(\omega_{k}t\right)+\varphi_{k}\left(t\right)\cos\left(\omega_{k}t\right).$$

At the output of the *k*-th phase detector, we obtain the following:

(6) $$sd_{k}\left(t\right)=\sin\left(\omega_{k}t\right)+\left(\varphi_{k}\left(t\right)+\varphi_{(k+1)\;\mathrm{mod}\;K}\left(t\right)\right)\cos\left(\omega_{k}t\right).$$

Next, signal $sd_{k}\left(t\right)$ is sampled. For simplicity, ideal sampling with Dirac pulses with $T_{s}$ period is assumed. The sampled signal is as follows:

(7) $$sd_{\delta_{k}}\left(t\right)=\sum_{n=-\infty }^{\infty }\left[\sin\left(\omega_{k}nT_{s}\right)+\left(\varphi_{k}\left(nT_{s}\right)+\varphi_{(k+1)\;\mathrm{mod}\;K}\left(nT_{s}\right)\right)\cos\left(\omega_{k}nT_{s}\right)\right]\delta \left(t-nT_{s}\right).$$

After sampling, the signals are combined modulo 2. Because the jitter model is linear [36], the phase fluctuations from each ring oscillator propagate to the output as a sum. Thus, the signal after combining XOR of *K* outputs of the phase detectors is given as follows:

(8) $$\begin{array}{c} s_{⊕}\left(t\right)=\sum_{n=-\infty }^{\infty }\sum_{k=0}^{K-1}\left[\sin\left(\omega_{k}nT_{s}\right)+\left(\varphi_{k}\left(nT_{s}\right)+\right.\right.\\ \left.\left.\varphi_{(k+1)\;\mathrm{mod}\;K}\left(nT_{s}\right)\right)\cos\left(\omega_{k}nT_{s}\right)\right]\delta \left(t-nT_{s}\right)-C, \end{array}$$

where *C* is a constant. Using the Fourier transform, the spectrum at the output of the generator is as follows:

(9) $$\begin{array}{c} S_{⊕}\left(\omega \right)=\frac{1}{2T_{s}}\sum_{n=-\infty }^{\infty }\sum_{k=0}^{K-1}\frac{1}{j}\left(\delta \left(\omega -\omega_{k}-n\omega_{s}\right)-\right.\\ \left.\delta \left(\omega +\omega_{k}-n\omega_{s}\right)\right)+\Phi_{k}\left(\omega -\omega_{k}-n\omega_{s}\right)+\\ \Phi_{k}\left(\omega +\omega_{k}-n\omega_{s}\right)+\Phi_{(k+1)\;\mathrm{mod}\;K}\left(\omega -\omega_{k}-n\omega_{s}\right)+\\ \Phi_{(k+1)\;\mathrm{mod}\;K}\left(\omega +\omega_{k}-n\omega_{s}\right)-2\pi C\delta \left(0\right). \end{array}$$

For comparison, the spectrum of the signal from a generator without a phase detector [4] is as follows:

(10) $$\begin{array}{c} Sw_{⊕}\left(\omega \right)=\frac{1}{2T_{s}}\sum_{n=-\infty }^{\infty }\sum_{k=0}^{K-1}\frac{1}{j}\left(\delta (\omega -\omega_{k}-n\omega_{s})-\right.\\ \left.\delta (\omega +\omega_{k}-n\omega_{s})\right)+\Phi_{k}\left(\omega -\omega_{k}-n\omega_{s}\right)+\\ \Phi_{k}\left(\omega +\omega_{k}-n\omega_{s}\right)-2\pi C\delta \left(0\right). \end{array}$$

**Figure 6:**
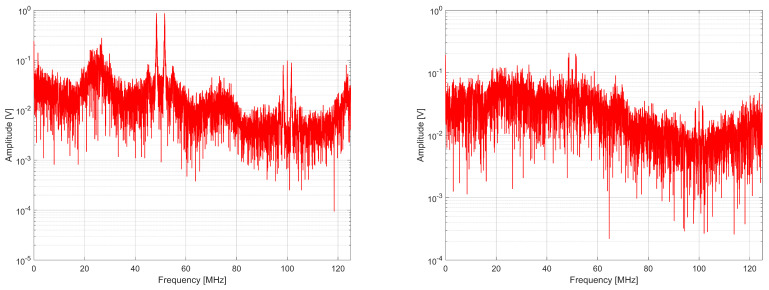
Comparison of the spectrum on the output of RO-based generator without phase detector (**left**) and with phase detector (**right**) for K = 2.

**Figure 7:**
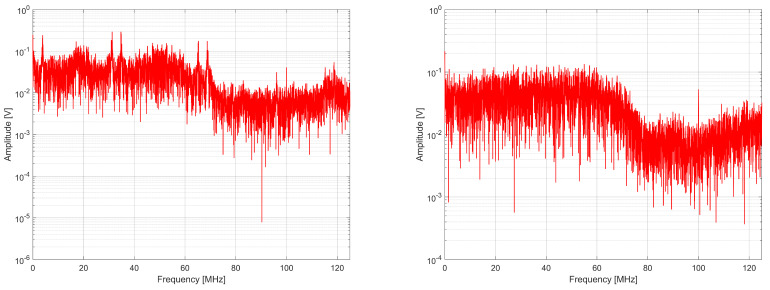
Comparison of the spectrum on the output of RO-based generator without phase detector (**left**) and with phase detector (**right**) for K = 4.

**Figure 8:**
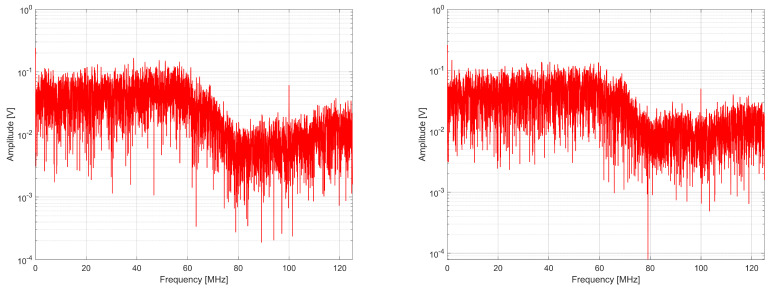
Comparison of the spectrum on the output of RO-based generator without phase detector (**left**) and with phase detector (**right**) for K = 8.

When analyzing Equations (9) and (10), it can be seen that in the proposed generator, the spectrum components corresponding to phase fluctuations from the *k*-th ring oscillator propagate to the output twice on two different subcarriers. This is desirable because it allows the band to be covered more accurately and likens the signal to white noise, as illustrated in Figure 6, Figure 7 and Figure 8. The tested generators consisted of K=2, 4, and 8 source ring oscillators. The sampling frequency was 100 MHz, and the bit rate was 100 Mbit/s.

When analyzing the spectra, it can be noted that the spectrum is centralized around several frequencies for the generator from [4]. In comparison, the spectrum of the generator with a phase detector is more diffused and covers the entire signal band better. This is confirmed by the values of spectral flatness determined using Wiener entropy described by the following formula [37]:

(11) $$F_{l}=\frac{\sqrt[{R}]{\prod_{r=0}^{R-1}x\left(r\right)}}{\frac{1}{R}\sum_{r=0}^{R-1}x\left(r\right)}=\frac{e^{\frac{1}{R}\sum_{r=0}^{R-1}\ln x\left(r\right)}}{\frac{1}{R}\sum_{r=0}^{R-1}x\left(r\right)},$$

where x(r) are the samples of the spectrum in the set *R* of samples (Table 3). A spectrum flatness of one means that we are dealing with white noise in the measured signal band, whereas the $F_{l}$ value equal to zero means pure harmonic.

The generator from Figure 4 was implemented in different devices from the main vendors on the market. Xilinx, Altera (Intel), and Lattice cover approximately 90% of the FPGA market. The FPGAs used in the tests were from Xilinx (Spartan-3 XC3S500E, Spartan-6 XC6LX16, Virtex-4 XC4VLX25, Virtex-5 XC5VLX50T, Virtex-6 XC6VLX240T, and Artix-7 XC7A35T), from Altera (Intel) (Cyclone-II EP2C35, Cyclone-IV EP4CE22, Cyclone-V 5CSEMA5, Cyclone-10 10CL025, Stratix-IV EP4SGX530, and MAX-10 10M50DA), and from Lattice (ECP3 LFE3-35EA-8FN484C, and ECP5 LFE5UM-45F-8BG381C). It is important to note that these devices are constructed using different technologies.

## 3. The Quality of Bit Streams Generated by the Proposed Generator

The statistical tests outlined in the document “A Statistical Test Suite for Random and Pseudo-Random Number Generators for Cryptographic Applications” (NIST SP 800-22) [38] were employed to evaluate the statistical quality of binary streams generated by a combined RNG utilizing ring oscillators and phase detectors. This test suite comprises 15 distinct statistical tests, several of which include multiple subtests. In this study, we focused on the worst-case scenario by selecting the least favorable outcome from the results of the various subtests.

The NIST has proposed two approaches for testing. The first approach involves examining the proportion $R_{\beta }$ of sequences that successfully pass a statistical test. In contrast, the second approach assesses the distribution of *p*-values computed by the software, focusing on the value of $P_{T}$ [38]. In this study, we adopted a standard set of parameters as recommended by the NIST in version 2.1.1. For a significance level of β=0.01 and 1000 tested sequences, each with a length of $10^{6}$ bits, the minimal passing proportion was determined to be approximately $R_{\beta }=0.9805$. Sequences were considered uniformly distributed if $P_{T}\geq 0.0001$. The NIST tests were conducted using K=10,15,20,25, and 30 elementary generators, with sampling frequencies $f_{L}=50$ MHz, 100 MHz, 200 MHz, and 300 MHz. The worst results were observed at $f_{L}=300$ MHz, regardless of the *K* value. Statistical tests were satisfied for K≥15 ROs, independent of the $f_{L}$ value and the FPGA vendor. All configurations passed all tests for K≥12 ROs at sampling frequencies of $f_{L}=50$ MHz or $f_{L}=100$ MHz.

Statistical tests from the NIST Recommendation 800-90B [39] were utilized to evaluate whether the generator produces independent bits with an identical distribution. The NIST’s Recommendation 800-90B proposes a permutation-based testing strategy. This approach tests a statistical hypothesis by comparing the actual value of the statistic with the reference distribution inferred from the input data. The general methodology involves generating 10,000 permutations from the input dataset, computing the test statistics for each permutation, and comparing these results with the test statistic calculated on the original dataset. This process is repeated for each statistical test. If the string elements are independent and identically distributed (IID), the permutation of the dataset should not significantly alter the value of the test statistic. Specifically, the original dataset and its permutations are expected to originate from the same distribution, leading to similar statistics. Thus, extremely high or low test results should be rare. Conversely, if the samples are not IID, the original and permuted test statistics may vary significantly. To determine the outcome of the test based on permutations, counters denoted as $C_{i,0}$ and $C_{i,1}$ are employed [39]. Extreme values of these counters indicate that the data samples are not IID. If the sum of $C_{i,0}$ and $C_{i,1}$ is less than 5, it suggests that the original test statistic has a very high rank. In contrast, if $C_{i,0}$ exceeds 9995, the original test statistic ranks very low. The cut-off values for $C_{i,0}$ and $C_{i,1}$ are set at a significance level of β=0.001. These NIST 800-90B tests were also conducted for K=10,15,20,25,30 elementary generators and sampling frequencies $f_{L}=50$ MHz, 100 MHz, 200 MHz, and 300 MHz. The results indicate that using K=10 elementary generators is sufficient to conclude that the source of randomness produces IID sequences. The worst-case minimal entropy value $H_{min}$ was approximately 0.984086. It is important to note that to achieve IID sequences at the generator’s output from [4], it was necessary to utilize more than 18 source ROs, resulting in a minimal entropy of about 0.832156.

The NIST 800-90B tests primarily indicate whether sequences are IID or non-IID, and the results of the embedded restart mechanism only reveal whether these tests have been passed. Therefore, an alternative approach utilizing a restart mechanism was employed to assess the influence of deterministic components on bit generation. During each restart, M=20,000 bits were generated, with a total of N=2048 restarts performed, consistent with the methodology in [29]. Subsequently, *M* chi-square tests were conducted on the *N*-bit sequences, with a significance level set at 0.01.

If among the 20,000 sequences, 1 or 2 consecutive sequences are found to fail the chi-square test, it is considered that a perfect random source may produce such results. This assumption is predicated on the notion that a perfect random number generator yields equally probable sequences, implying that the occurrence of several consecutive failures in the chi-square test should be infrequent. The expected number of possible consecutive failures can be computed [29], with the exact value dependent on *N* and the chosen significance level of the chi-square test.

In the event that 2 consecutive sequences fail the chi-square test among the 20,000 generated sequences, this source is not disqualified as a generator of true randomness. However, if the number of consecutive failures exceeds two, it can be concluded that the sequences are not produced by a perfect random source, as three consecutive failures statistically occur approximately every $10^{-6}$ sequences, significantly exceeding the size *M* of the probe [29].

A dedicated algorithm is employed to search for the maximum *m* such that 3≤m≤20,000, for which the 2048-bit sequences generated for *m*, m−1, and m−2 fail the chi-square test. For j>m, there is no justification for rejecting the hypothesis that a non-deterministic source generates the elements of the 2048-bit sequences. The smallest *j*, equal to m+1, is denoted as $m_{min}$. If three consecutive failures of the chi-square test are not found, it is assumed that $m_{min}=1$. This restart mechanism effectively illustrates the generator’s run-up. It is crucial to ascertain how many bits exhibit bias when the generator commences operation, as this may result in the generated random key possessing significantly fewer random bit states than anticipated.

**Figure 9:**
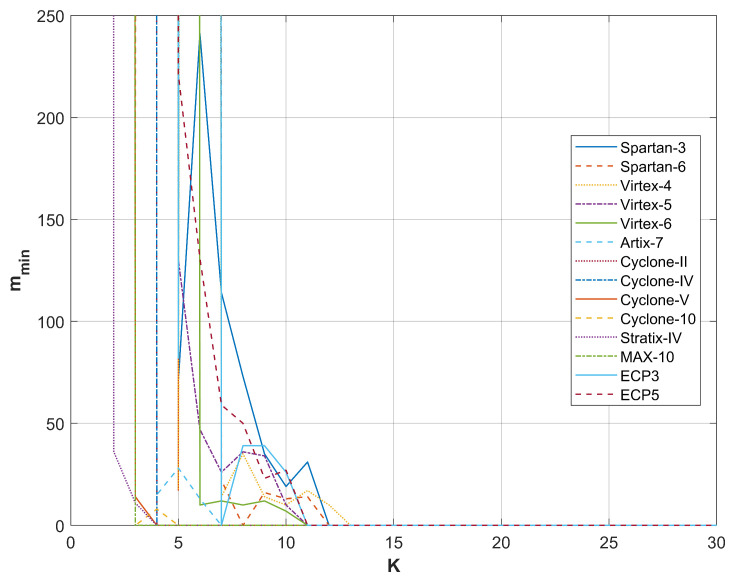
$m_{min}$ as a function of *K* for sampling frequency $f_{L}=50$ MHz.

**Figure 10:**
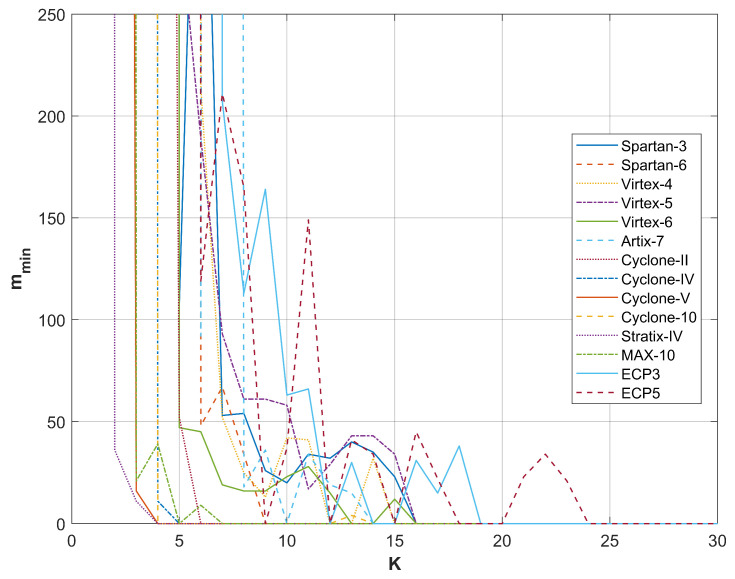
$m_{min}$ as a function of *K* for sampling frequency $f_{L}=100$ MHz.

**Figure 11:**
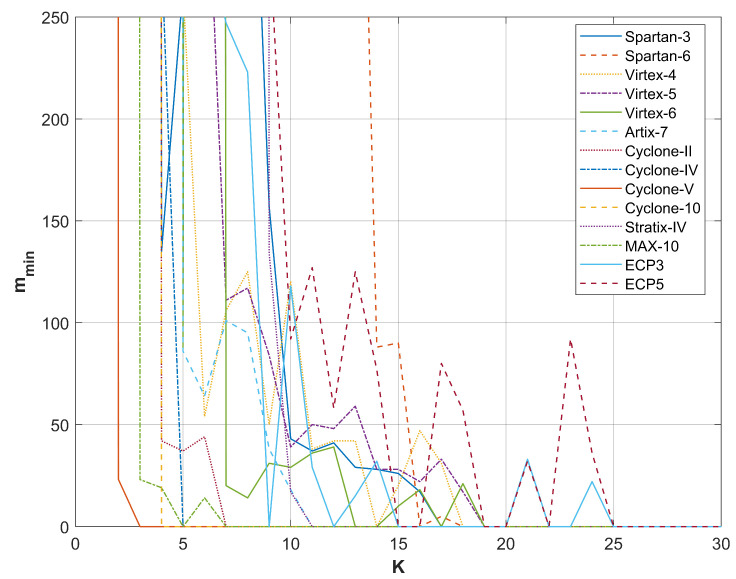
$m_{min}$ as a function of *K* for sampling frequency $f_{L}=200$ MHz.

**Figure 12:**
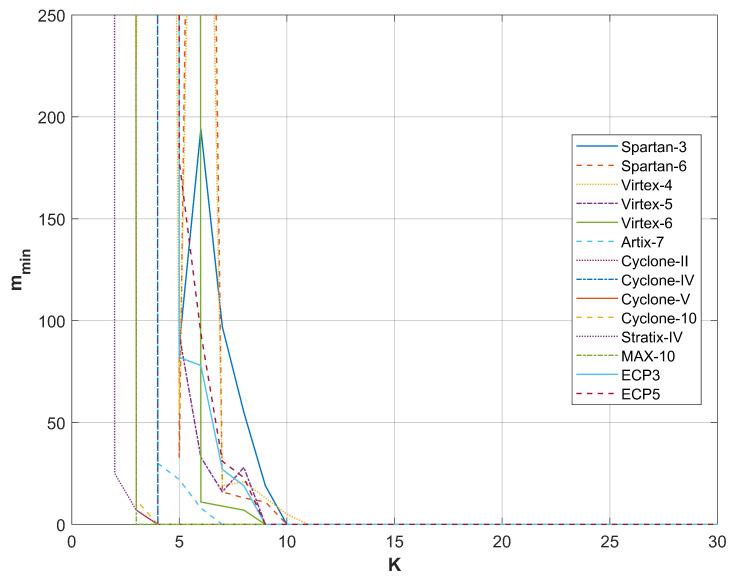
$m_{min}$ as a function of *K* for sampling frequency $f_{L}=300$ MHz.

A comparison of the results from restarts for different FPGA devices producing random bits with the RO-based combined RNG utilizing a phase detector is presented in Figure 9, Figure 10, Figure 11 and Figure 12. For all sampling frequencies, the value of $m_{min}$ decreases irregularly with an increase in *K*. By combining XOR bits from multiple elementary generators, the amount of harvested randomness is enhanced. For large values of *K*, $m_{min}$ approaches one, indicating that all bits in the *M*-bit sequences are generated as a result of a non-deterministic process.

The parameter *K* at which $m_{min}$ reaches one increases with the sampling frequency $f_{L}$; it is approximately 9 for $f_{L}=50$ MHz, 13 for $f_{L}=100$ MHz, 20 for $f_{L}=200$ MHz, and 25 for $f_{L}=300$ MHz. Table 4 presents the resources necessary to achieve $m_{min}=1$ and to pass all NIST statistical tests for the output sequences corresponding to the analyzed sampling frequencies. The values presented in Table 4 indicate that the RNG with the phase detector requires significantly fewer resources than the RNG without the phase detector. Furthermore, the disparity in resource requirements increases with the rise in sampling frequency $f_{L}$. Table 5 shows the results of NIST 800-90B tests for the same generators as in Table 4.

## 4. Optimization of Resources

The output sequences pass the NIST statistical tests for K≥15 across all sampling frequencies and for all vendors of FPGAs. However, to produce an unbiased *N*-bit sequence, at least 25 elementary generators are required when $f_{L}=300$ MHz. This discrepancy in *K* values indicates that the fundamental limitation for generating random bits at high bit rates is not the number of elementary generators necessary to pass the NIST tests but rather the amount of harvested randomness. This issue is pertinent to both classic constructions and the RNG proposed in this paper.

**Figure 13:**
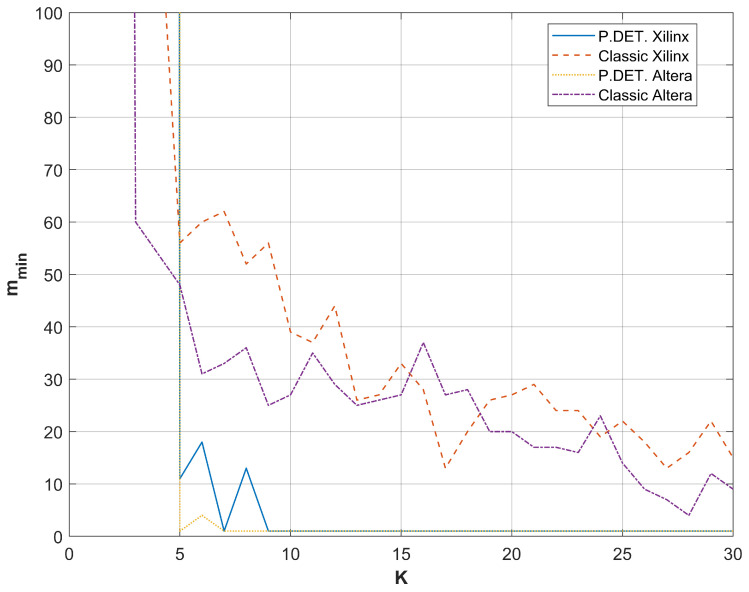
Comparison of $m_{min}$ values as a function of *K* for two vendors of FPGAs and two solutions of RNG: classic and with additional phase detectors. The sampling frequency was set to 50 MHz.

To establish a balance between *K*, which ensures sufficient statistical quality, and *K*, which yields an adequate amount of randomness, sampling frequencies were reduced below 50 MHz. A rapid decline in the $m_{min}$ value was observed for both the classic RNG and the RNG utilizing additional phase detectors (see Figure 13). Meanwhile, no proportional reduction in *K* necessary to pass the statistical tests was noted. This observation supports the thesis that the favorable statistical properties observed in many RO-based RNGs may arise from the coexistence of deterministic and non-deterministic components in the output bit sequence. As *K* increased, the proportion of these components changed. For K≥25, the non-deterministic component predominated, ensuring $m_{min}=1$. Therefore, when focusing solely on the statistical tests of output sequences produced by RO-based RNGs, a misleading conclusion about the randomness of the RNG may be drawn, as first noted in [27].

Further experiments demonstrated that $m_{min}$ achieved 1 for the RNG with phase detectors, whereas the classic construction yielded an $m_{min}$ value of approximately 30 (the exact value varied depending on the FPGA type) for the same *K* and the same sampling frequency of 50 MHz. At a sampling frequency of 25 MHz, $m_{min}=1$ was obtained with seven ROs for the RNG with a phase detector, while $m_{min}=9$ was observed for the RNG described in [4]. For $f_{L}=10$ MHz, $m_{min}=1$ was reached with six ROs, corresponding to $m_{min}=6$ for the RNG from [4]. At $f_{L}=1$ MHz, $m_{min}=1$ was achieved with four ROs, which also yielded $m_{min}=1$ for the RNG from [4].

Thus, at relatively low sampling frequencies, both the proposed generator and the RNG described in [4] provided comparable amounts of randomness. However, the minimum values of *K* required to ensure the high statistical quality of the output bit streams differed. For the proposed RNG, K=7 elementary generators were needed for sampling frequencies not exceeding 25 MHz. In contrast, the same RNG without phase detectors required K=10, but the sampling frequency could not exceed 1 MHz. In both cases, $m_{min}=1$.

Table 6 compares the FPGA resources utilized by the proposed RNG optimized for *K* with the resources required to implement a classic RNG that delivers the same bit rate. In both cases, $m_{min}=1$, and the output sequences passed the NIST statistical tests for all FPGA vendors examined in this paper.

## 5. Conclusions

In this paper, a novel method for producing high-entropy random numbers is proposed. This method utilizes the concept of a combined RNG, augmented with additional phase detectors, to add entropy from the phase jitter of ring oscillators. The proposed RNG generates bits on demand and can be easily integrated into a single FPGA or ASIC within any digital system that requires random bits. By increasing the number of elementary RNGs to at least K=25, random bits can be produced at a rate of 300 Mbit/s for any FPGA manufacturer.

The areas of application encompass all systems that necessitate high-entropy random bits, with the proposed RNG being particularly suited for cryptographic applications. The generator utilizes less than 1% of the resources available in the smallest FPGA device and consumes less than 100 mW across all sampling frequencies, with *K* not exceeding 50. For security reasons, it is advisable to implement more than K=25 elementary generators when $f_{L}=300$ MHz and to use more than seven generators for the optimized version operating at a sampling frequency of 25 MHz. A greater *K* enhances the uniformity of the distribution of oscillator frequencies, thereby increasing the robustness of the RO-based RNG against frequency injection attacks.

## Figures and Tables

**Table 1:** The truth table of the phase detector.

*J*	*K*	$Q_{n+1}$
0	0	$Q_{n}$
0	1	0
1	0	1
1	1	$\bar{Q_{n}}$

**Table 2:** Frequencies of ring oscillators.

K	Spartan-3 [MHz]	Spartan-6 [MHz]	Artix-7 [MHz]	Cyclone-V [MHz]	Max-10 [MHz]
1	366.4	444.8	583.2	295.6	839.2
2	361.2	466.2	393.8	454.6	675.2
3	361.8	443.4	336.4	1220.6	850.0
4	377.4	473.8	473.4	786.8	563.6
5	281.4	456.4	339.8	419.0	898.2
6	371.4	455.0	339.4	325.8	933.8
7	372.2	442.0	489.6	516.8	676.0
8	370.6	436.0	653.4	819.0	938.2
9	376.8	467.4	313.4	795.0	946.6
10	374.2	458.0	415.6	436.6	816.8
11	287.6	437.6	493.6	409.6	854.8
12	281.2	469.4	338.8	366.4	852.4
13	376.0	462.4	338.8	311.2	857.8
14	294.0	465.8	489.6	402.6	826.6
15	282.4	480.0	386.2	863.8	701.6
16	381.0	479.8	558.6	420.6	880.2
17	360.2	466.0	368.2	309.8	849.0
18	369.4	485.2	474.4	753.2	699.0
19	372.6	468.6	540.4	433.2	850.4
20	288.4	459.8	378.0	1255.4	717.2
21	361.6	479.2	340.8	1247.4	860.0
22	376.8	452.6	501.6	311.2	847.8
23	233.2	465.2	406.0	1196.4	718.2
24	367.8	463.0	378.6	488.8	729.8
25	281.0	465.6	346.6	1326.8	782.2
26	380.0	440.8	371.2	315.0	817.4
27	368.0	478.0	417.8	508.6	852.2
28	275.8	444.2	383.8	551.4	525.2
29	291.0	479.2	317.6	1434.6	833.0
30	375.0	487.6	415.4	333.6	892.0
AVG.	340.5	462.4	419.5	643.6	802.8
STD.	45.2	14.7	87.3	364.0	103.9
Δf	147.8	51.6	340.0	1139.0	421.4
$\Delta f=f_{max}-f_{min}$					

**Table 3:** Spectral flatness comparison of RO-based RNGs.

	RNG from [4]	RNG with Phase Detector
K = 2	0.553671	0.673340
K = 4	0.599396	0.700494
K = 8	0.651399	0.707224

**Table 4:** Resources used by the proposed RNG in comparison to RNG from [4] for $m_{min}=1$.

	RNG with Phase Detector				RNG from [4]			
$f_{s}\;\left[MHz\right]$	50	100	200	300	50	100	200	300
*K*	9	13	20	25	43	79	146	208
$m_{min}$	1	1	1	1	1	1	1	1
Slices	21	40	75	84	50	92	184	307
Slices LUTs	38	72	120	157	136	252	467	665
Slices registers	23	43	61	96	57	106	194	286

**Table 5:** Resources used by the proposed RNG in comparison to RNG from [4] for $m_{min}=1$.

	RNG with Phase Detector				RNG from [4]			
$f_{s}\;\left[MHz\right]$	50	100	200	300	50	100	200	300
*K*	9	13	20	25	43	79	146	208
IID	NO	YES	YES	YES	YES	YES	YES	YES
$H_{min}$	0.9838	0.9950	0.9958	0.9959	0.9927	0.9938	0.9949	0.9960

**Table 6:** Resources used by the optimized RNG with phase detector in comparison to RNG from [4].

	RNG with Phase Detector	RNG from [4]
$f_{s}$ [MHz]	25	25
*K*	7	27
$m_{min}$	1	1
IID	NO	YES
$H_{min}$	0.800764	0.994086
Slices	17	31
Slices LUTs	30	73
Slices registers	18	36

## Data Availability

The original contributions presented in this study are included in the article. Further inquiries can be directed to the corresponding author.
